# Ten Simple Rules for Making Good Oral Presentations

**DOI:** 10.1371/journal.pcbi.0030077

**Published:** 2007-04-27

**Authors:** Philip E Bourne

Continuing our “Ten Simple Rules” series [1–5], we consider here what it takes to make a good oral presentation. While the rules apply broadly across disciplines, they are certainly important from the perspective of this readership. Clear and logical delivery of your ideas and scientific results is an important component of a successful scientific career. Presentations encourage broader dissemination of your work and highlight work that may not receive attention in written form.

## Rule 1: Talk to the Audience

We do not mean face the audience, although gaining eye contact with as many people as possible when you present is important since it adds a level of intimacy and comfort to the presentation. We mean prepare presentations that address the target audience. Be sure you know who your audience is—what are their backgrounds and knowledge level of the material you are presenting and what they are hoping to get out of the presentation? Off-topic presentations are usually boring and will not endear you to the audience. Deliver what the audience wants to hear.

## Rule 2: Less is More

A common mistake of inexperienced presenters is to try to say too much. They feel the need to prove themselves by proving to the audience that they know a lot. As a result, the main message is often lost, and valuable question time is usually curtailed. Your knowledge of the subject is best expressed through a clear and concise presentation that is provocative and leads to a dialog during the question-and-answer session when the audience becomes active participants. At that point, your knowledge of the material will likely become clear. If you do not get any questions, then you have not been following the other rules. Most likely, your presentation was either incomprehensible or trite. A side effect of too much material is that you talk too quickly, another ingredient of a lost message.

## Rule 3: Only Talk When You Have Something to Say

Do not be overzealous about what you think you will have available to present when the time comes. Research never goes as fast as you would like. Remember the audience's time is precious and should not be abused by presentation of uninteresting preliminary material.

## Rule 4: Make the Take-Home Message Persistent

A good rule of thumb would seem to be that if you ask a member of the audience a week later about your presentation, they should be able to remember three points. If these are the key points you were trying to get across, you have done a good job. If they can remember any three points, but not the key points, then your emphasis was wrong. It is obvious what it means if they cannot recall three points!

## Rule 5: Be Logical

Think of the presentation as a story. There is a logical flow—a clear beginning, middle, and an end. You set the stage (beginning), you tell the story (middle), and you have a big finish (the end) where the take-home message is clearly understood.

## Rule 6: Treat the Floor as a Stage

Presentations should be entertaining, but do not overdo it and do know your limits. If you are not humorous by nature, do not try and be humorous. If you are not good at telling anecdotes, do not try and tell anecdotes, and so on. A good entertainer will captivate the audience and increase the likelihood of obeying Rule 4.

## Rule 7: Practice and Time Your Presentation

This is particularly important for inexperienced presenters. Even more important, when you give the presentation, stick to what you practice. It is common to deviate, and even worse to start presenting material that you know less about than the audience does. The more you practice, the less likely you will be to go off on tangents. Visual cues help here. The more presentations you give, the better you are going to get. In a scientific environment, take every opportunity to do journal club and become a teaching assistant if it allows you to present. An important talk should not be given for the first time to an audience of peers. You should have delivered it to your research collaborators who will be kinder and gentler but still point out obvious discrepancies. Laboratory group meetings are a fine forum for this.

## Rule 8: Use Visuals Sparingly but Effectively

Presenters have different styles of presenting. Some can captivate the audience with no visuals (rare); others require visual cues and in addition, depending on the material, may not be able to present a particular topic well without the appropriate visuals such as graphs and charts. Preparing good visual materials will be the subject of a further Ten Simple Rules. Rule 7 will help you to define the right number of visuals for a particular presentation. A useful rule of thumb for us is if you have more than one visual for each minute you are talking, you have too many and you will run over time. Obviously some visuals are quick, others take time to get the message across; again Rule 7 will help. Avoid reading the visual unless you wish to emphasize the point explicitly, the audience can read, too! The visual should support what you are saying either for emphasis or with data to prove the verbal point. Finally, do not overload the visual. Make the points few and clear.

## Rule 9: Review Audio and/or Video of Your Presentations

There is nothing more effective than listening to, or listening to and viewing, a presentation you have made. Violations of the other rules will become obvious. Seeing what is wrong is easy, correcting it the next time around is not. You will likely need to break bad habits that lead to the violation of the other rules. Work hard on breaking bad habits; it is important.

## Rule 10: Provide Appropriate Acknowledgments

People love to be acknowledged for their contributions. Having many gratuitous acknowledgements degrades the people who actually contributed. If you defy Rule 7, then you will not be able to acknowledge people and organizations appropriately, as you will run out of time. It is often appropriate to acknowledge people at the beginning or at the point of their contribution so that their contributions are very clear.

As a final word of caution, we have found that even in following the Ten Simple Rules (or perhaps thinking we are following them), the outcome of a presentation is not always guaranteed. Audience–presenter dynamics are hard to predict even though the metric of depth and intensity of questions and off-line followup provide excellent indicators. Sometimes you are sure a presentation will go well, and afterward you feel it did not go well. Other times you dread what the audience will think, and you come away pleased as punch. Such is life. As always, we welcome your comments on these Ten Simple Rules by Reader Response.
